# Benthic community structure, diversity, and productivity in the shallow Barents Sea bank (Svalbard Bank)

**DOI:** 10.1007/s00227-012-2135-y

**Published:** 2012-12-20

**Authors:** Monika Kędra, Paul E. Renaud, Hector Andrade, Ilona Goszczko, William G. Ambrose

**Affiliations:** 1 Institute of Oceanology, Polish Academy of Sciences, Powstańców Warszawy 55, 81-712 Sopot, Poland; 2 Present Address: Chesapeake Biological Laboratory Center for Environmental Science, University of Maryland, P.O. BOX 38, Solomons, MD 20 688 USA; 3 Akvaplan-niva, Fram Centre for Climate and the Environment, 9296 Tromsø, Norway; 4 University Centre on Svalbard, 9071 Longyearbyen, Norway; 5 Department of Biology, Bates College, Lewiston, ME 04240 USA

## Abstract

The Barents Sea is among the most productive areas in the world oceans, and its shallow banks exhibit particularly high rates of primary productivity reaching over 300 g C m^−2^year^−1^. Our study focused on the Svalbard Bank, an important feeding area for fishes and whales. In order to investigate how benthic community structure and benthic secondary production vary across environmental gradients and through time, we sampled across the bank and compared results with a similar study conducted 85 years ago. Considerable variability in community structure and function across bank corresponded with differences in the physical structure of the habitat, including currents, sedimentation regimes and sediment type, and overlying water masses. Despite an intensive scallop fishery and climatic shifts that have taken place since the last survey in the 1920s, benthic community structure was very similar to that from the previous survey, suggesting strong system resilience. Primary and secondary production over shallow banks plays a large role in the Barents Sea and may act as a carbon subsidy to surrounding fish populations, of which many are of commercial importance.

## Introduction

The Arctic Ocean and its marginal seas overlie more than 25 % of the world’s continental shelves, and many of these shallow areas are characterized by seasonally high primary production which settles rapidly to the benthos, supporting rich communities of fishes, birds, and mammals (Grebmeier et al. 2006). Studies of biotic processes on these shelves help identify the important links between pelagic and benthic systems, and the role of environmental factors such as depth, ocean currents, and climate variability on benthic community structure.

The fate of primary production in Arctic shelf seas depends upon numerous water-column processes related to spatial and temporal variability in vertical export, including feeding intensity of zooplankton in the upper layers (Wassmann and Reigstad 2011). Typically, about 44–67 % of primary production in the Barents Sea reaches the sea floor (Wassmann et al. 2006a, b). In Arctic systems, benthic faunal assemblages respond rapidly to deposition of primary production (e.g., McMahon et al. 2006; Renaud et al. 2008). Primary production is spatially heterogeneous and often occurs in the form of episodic pulses of pelagic- and ice-related organic carbon (Ambrose and Renaud 1997; Ambrose et al. 2005; Carmack and Wassmann 2006). This results in close relationships between food availability and macrofaunal abundance, community structure, biomass, biodiversity, and benthic carbon cycling (Grebmeier et al. 1988; Grebmeier and McRoy 1989; Renaud et al. 2007, 2008; Carroll et al. 2008; Cochrane et al. 2009, 2012) and gives a strong support to the paradigm of tight pelagic-benthic coupling on Arctic shelves (Grebmeier and Barry 1991; Piepenburg 2005). Our understanding of many of these links, however, remains qualitative. Quantitative data on carbon demand and benthic secondary production are crucial for parameterizing ecosystem models for further investigation of the role of the benthos in system functioning, now and in the future.

Benthic secondary productivity is one parameter that quantitatively links pelagic and benthic communities and at the same time can be a valuable parameter for regional comparisons of potential contribution to fisheries production. Additionally, the proportion of benthic production relative to primary productivity has been hypothesized as being greater at high latitudes due to tight pelagic-benthic coupling in Arctic and sub-Arctic seas (Brey and Clarke 1993; Cusson and Bourget 2005; Grebmeier et al. 2006). Consequently, these vital rates provide an important baseline for regional comparisons and assessment of possible system change.

The Barents Sea is one of the most productive marginal seas of the world’s oceans (Sakshaug and Slagstad 1991; Sakshaug 1997; Carmack and Wassmann 2006), with an estimated overall average annual primary productivity of about 100 g C m^−2^ year^−1^ (Sakshaug et al. 2009). The Barents Sea and Svalbard waters are highly productive provinces accounting for 49 % of the total pan-Arctic shelf primary production (Sakshaug 2004). Deeper (>200 m), depositional areas accumulate soft sediments, the infaunal inhabitants of which provide nutrition for economically valuable shrimp and demersal fish populations. Benthic community structure throughout much of the deeper Barents Sea has been well characterized (e.g., Zenkevich 1963; Dahle et al. 1998; Carroll et al. 2008; Cochrane et al. 2009), and there is some understanding of factors influencing benthic processes (Piepenburg et al. 1995; Renaud et al. 2008).

Less well-studied are shallow banks, which make up more than one-third of the area of the Barents Sea (Jakobsson 2002) and can exhibit high rates of primary productivity. The waters over Svalbard Bank, which at their shallowest are less than 40 m deep, are estimated to have primary production over 300 g C m^−2^ year^−1^ (Sakshaug et al. 2009), have supported a commercially viable scallop fishery, and represent an important feeding area for fish (Loeng and Drinkwate 2007), and whales (Skern-Mauritzen et al. 2011). Combined epi- and infauna analysis (dredge and grab sampling) is important in comprehensive marine environmental studies (Jørgensen et al. 2011). However, the last detailed combined epifaunal and infaunal survey of Svalbard Bank was conducted in 1925 (Idelson 1930). In the 85 years between that study and the present investigation, the area has seen fluctuating periods of warming (1930s–1950s) and cooling (1960s–1980s), an intense scallop fishery (1987–1992), and the current extended period of climate warming. All of these events may well have influenced seafloor communities (e.g., Blacker 1965). Prolonged warming and its predicted consequences for primary production (Ellingsen et al. 2008) are likely to continue to affect the structure and function of marine benthos, which may further affect predators such as shrimp, fishes, birds, and large mammals. Thus, a comparison of current community structure with historical data provides information on the resilience/resistance of these shallow benthic communities to a variety of potential agents of ecological change and may provide a model for other shallow banks which are common features on the shelf of the Arctic Ocean.

In an effort to fill some of these important knowledge gaps, we investigated how benthic densities, biomass, diversity, community structure, and productivity vary across depth and water mass gradients over the Svalbard Bank. We compared these data with those from a similar study conducted more than 80 years previously. Finally, we provide the first estimates of benthic secondary production and carbon demand from the Barents Sea. These results have implications for ecosystem resilience and carbon cycling and provide important data for future studies of temporal fluctuations in benthic fauna and ecosystem functioning.

## Materials and methods

### Study area

**Fig. 1:**
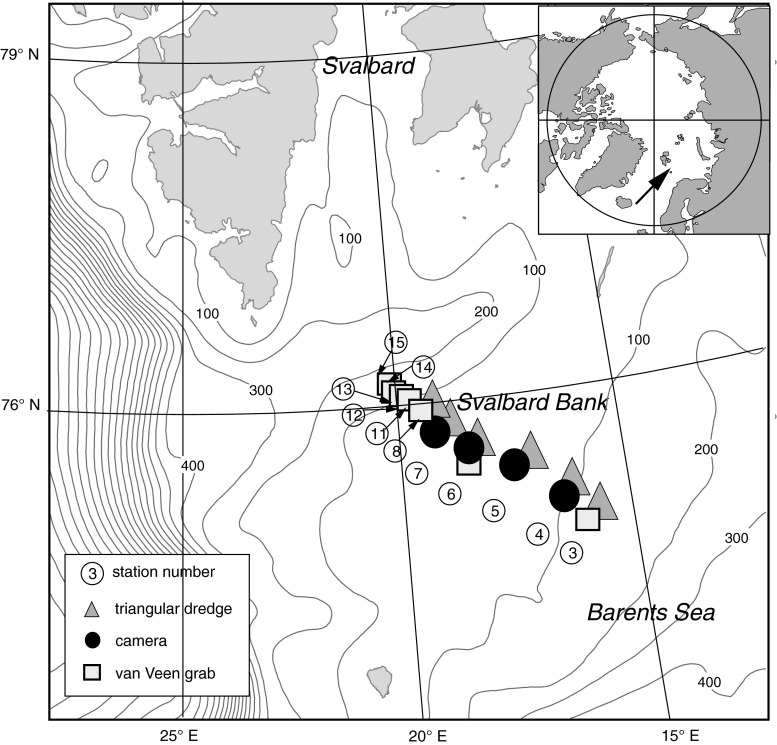
Sampling locations across Svalbard Bank

The Svalbard Bank is a shallow area in the western Barents Sea with a minimum depth of less than 40 m (Fig. 1). It is the largest open-shelf cold-water carbonate platform in the Arctic, built from barnacle sands (*Balanus balanus, B. crenatus*), mollusk shell fragments (*Mya truncata, Hiatella arctica,* and *Chlamys islandica*), and mixed with very coarse sand and gravel (Elverhøi and Solheim 1983; Henrich et al. 1997). On the slopes, the sediment composition varies from gravel and boulders, to mud and silt (Elverhøi and Solheim 1983; Henrich et al. 1997). In winter and spring, water over the bank is usually ice covered (Shapiro et al. 2003) while the proximity of Atlantic water keeps the southern slope ice-free throughout the year. Even north of the Polar Front, sea ice is easily advected by winds, and large areas may open up in a relatively short time, potentially leading to ice-edge blooms and high new production to the north of the Polar Front when light is available (Sakshaug 1997). The entire bank area is dominated by strong tidal currents and pronounced vertical mixing (Midttun 1985; Anderson et al. 1988; Kowalik and Proshutinsky 1995; Schauer 1995), which keeps the water column at the bank top mixed from surface to bottom throughout the year. Consequently, Svalbard Bank is the most productive area in the Barents Sea (Slagstad and McClimans 2005; Carmack and Wassmann 2006; Wassmann et al. 2006a), with annual productivity estimates over 300 g C m^−2^ year^−1^ (Sakshaug et al. 2009) and one of the most productive in the arctic marginal seas.

### Sampling

**Fig. 2:**
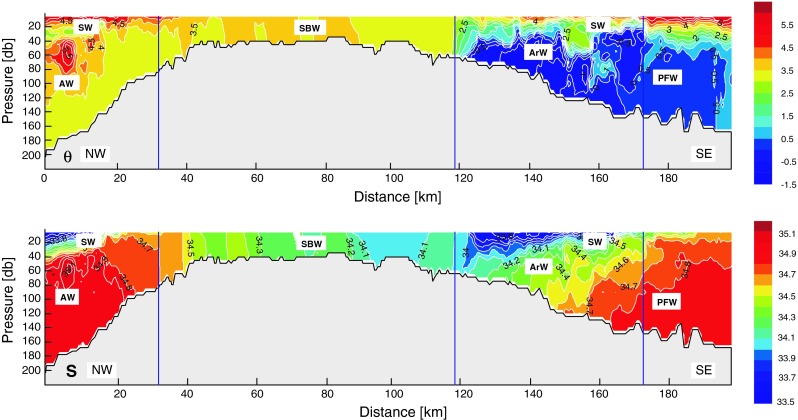
Temperature (θ) and salinity (S) distribution on the section across the Svalbard Bank during the r/v Oceania 2009 summer cruise. Four individual parts were separated to analyze water masses distribution: the northwestern Atlantic-dominated part, the middle shallow part, Arctic-dominated Polar Front zone, and Atlantic-influenced southeastern part (from left to the right). The major water masses: Atlantic Water (AW), Arctic Water (ArW), Svalbard Bank Water (SBW), Surface Water (SW), and Polar Front Water (PFW) are marked. *NW* northwest and *SE* southeast directions are shown. Modified from Węsławski et al. (2012)

A 200-km-long hydrographic transect was made between August 9 and 10, 2009, aboard r/v Oceania with the “tow-yo” Sea-Bird Electronics, Inc. (SBE) 49FastCAT CTD Sensor, cycling up and down through the water column, starting from the southern bank of the Storfjord Trough in the northwest, crossing the shallow bank top, and reaching the northern slope of the Bear Island Trough in the southeast. In total, data from 320 up and down casts were collected. Based on these measurements the distribution of main physical parameters (potential temperature and salinity) was drawn along the section (Fig. 2, modified from Węsławski et al. 2012), and the main water masses were identified. Water masses were classified based on a modified version of the criteria employed by Loeng (1991), Hopkins (1991), and Harris et al. (1998).

Faunal material was collected during the same cruise from 11 stations located across the Svalbard Bank at depths varying from 40 to 150 m. A van Veen grab (0.1 m^2^ sample area) was used to collect infaunal samples whenever possible from the east and west bank slopes, resulting in 26 samples from seven stations (Fig. 1; Table 1). At some stations, due to the sediment properties (station 6) and bad weather (station 15), it was not possible to obtain five grab replicates (Table 1). Material was sieved onboard through 0.5-mm mesh and fixed in buffered 10 % formalin. Later in the laboratory, macrofauna was sorted, counted, weighed (wet formalin weight), and identified to the lowest possible taxonomic level. To obtain estimates of epifaunal density and organisms sizes, an underwater “bottom-looking” drop camera was deployed at four stations at the top of the bank, and digital video footage was recorded from three replicate transects per stations (Fig. 1; Table 1). Fifteen-minute video transects were taken at epifaunal stations during ship drift. In all, 10 video records from stations 4–7 were analyzed in detail using frame captures approximately every 10 s (*n* = 40 − 125 frames per transect). To complement underwater video information, epifauna was collected at the same transect stations with a triangular dredge (1 m on each side; Table 1). Fauna collected by dredge was identified, measured (a selection of different sizes of organisms was made), and weighed. For each species a size–biomass relationship was established. On the basis of supplemental information gathered by the dredge, organisms on the video were identified to the lowest possible taxonomic level, counted from each snapshot, and measured. Biomass was determined later using the empirical relationships calculated from dredge samples.

**Table 1:** Sampling effort and basic station information on samples used in the present study

Station	Sampling gear	Location	Depth [m]	Replicates	Sediment type
3	Dredge/grab	75° 21 24° 09	137	3/4	Mud and gravel
4	Dredge/camera	75° 25 23° 35	95	3/3	Stones, gravel, and mud
5	Dredge/camera	75° 34 22° 35	65	3/3	Stones and gravel
6	Dredge/camera/grab	75° 44 21° 38	40	3/3/1	Shells, stones, and gravel
7	Dredge/camera	75° 50 20° 47	43	3/3	Shells, stones, and gravel
8	Dredge/grab	76° 00 20° 00	100	3/5	Stones, gravel, and mud
11	Grab	75° 58 20° 13	94	5	Mud and gravel
12	Grab	75° 59 20° 03	103	5	Mud and gravel
13	Grab	76° 01 19° 56	113	5	Mud and gravel
14	Grab	76° 01 19° 52	133	5	Mud
15	Grab	76° 02 19° 46	150	2	Mud

Where more than one gear is used, the number of replicates is given for each gear type

### Conversions and calculation of P/B ratio

To convert biomass (dry mass/m^2^, g) into energy (KJ) and thereafter to production values (KJ/year), data were first transformed using published conversion factors (Brey 2001). Subsequently, P/B ratios were calculated by employing a multiple regression model incorporating habitat (e.g., water temperature, depth, etc.)- and taxon (e.g., phylum level, motility)-specific data (Brey 2001; Bolam et al. 2010). For the video samples where the size of each organism was estimated, a P/B ratio was calculated for up to three size categories (large, medium, and small) for each species, depending upon how much the P/B ratio varied with organism size. To calculate secondary production, the biomass per area of each organism was multiplied by the size-dependent P/B ratio. Total production values for each replicate were then calculated as the sum of production values for each individual aggregated at the phylum level of taxonomic resolution. Production at each station was represented as the average of the replicates. For the grab samples, production values were similarly calculated for each organism and again, aggregated at the phylum level (i.e., calculated production values for all individuals within a phylum were summed). Finally, average production values were transformed to carbon using the conversion factor 45.7 J = 1 mg C (Salonen et al. 1979), and all data were standardized to a per m^2^ basis.

### Data analysis

Nonmetric Multidimensional Scaling (nMDS) of Bray–Curtis similarities, computed after fourth-root transformation of taxa-level abundance data, was conducted. The term Community Structure, as later used in the paper, encompasses species richness, density, and biomass. We tested for differences in community composition among stations using one-way ANOSIM permutation tests of the Bray–Curtis similarity data (Clarke and Warwick 2001). Species richness (S—number of species in sample) was calculated for each sample. Differences in density, species richness, biomass, and productivity among stations were tested with the use of the nonparametric Kruskal–Wallis test and Dunn’s post hoc multiple comparisons test.

The data analyses were performed using the PRIMER package v. 6 (Clarke and Warwick 2001) and the Statsoft software STATISTICA v. 6. CTD data were first checked and corrected by hand and then processed in the SBE Data Processing Software. Profiles were vertically averaged with 5-dbar intervals. Further calculations and visualization were developed in MathWorks Matlab environment.

## Results

### Hydrography

The northwestern part of the section deeper than 40 m was occupied by warm and salty Atlantic Water (AW) originating from the West Spitsbergen Current, distinguishable as a subsurface maximum of temperature and salinity. The surface layer was warm, about 33.5 PSU Surface Water (SW), which forms in summer due to solar-driven ice melting. The southeast part of the section was the Polar Front location where cold and low-saline Arctic Water (ArW) coming from the Arctic Ocean east of Svalbard was present. In the deeper layer, however, this water was subducted by saltier and slightly warmer water produced from AW transformation during mixing and cooling in the Barents Sea. The transition state between them is the Polar Front Water—saltier than ArW but colder than AW. The surface layer was occupied by warm and fresh SW, similarly as in the northwestern part of the section. In the middle, shallow part of the Svalbard Bank, water was well mixed due to strong tidal and wind forcing, forming a local water mass—Svalbard Bank Water (SBW)—that was relatively warm and with lower salinity (Fig. 2).

### Benthic epi- and infaunal characteristics

**Fig. 3:**
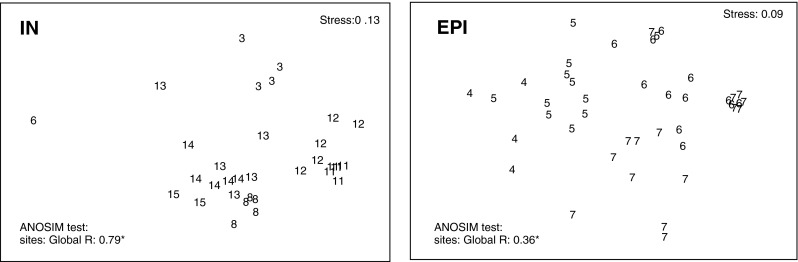
Nonmetric Multidimensional Scaling (nMDS) of Bray–Curtis similarities of fourth-root-transformed abundance data of benthos: *IN* infauna and *EPI* epifauna across Svalbard Bank. The results of ANOSIM tests for differences between sampling depths are also presented on the plot. Significant test results are marked with *, and pairwise differences are discussed in “Results”

**Fig. 4:**
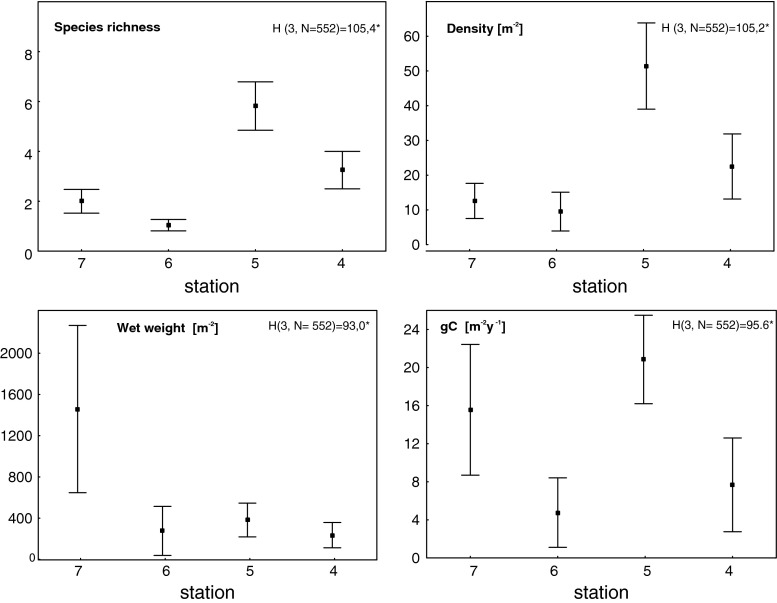
Mean epifauna species richness, density [number of individuals per m^−2^], wet weight [g m^−2^], and secondary production [g C m^−2^year^−1^] for each station with 0.95 confidence intervals. Kruskal–Wallis results for differences between sampling sites are given with significant test results marked with *. Stations arranged from northwest (*left*) to southeast (*right*) on the *x*-axis

**Fig. 5:**
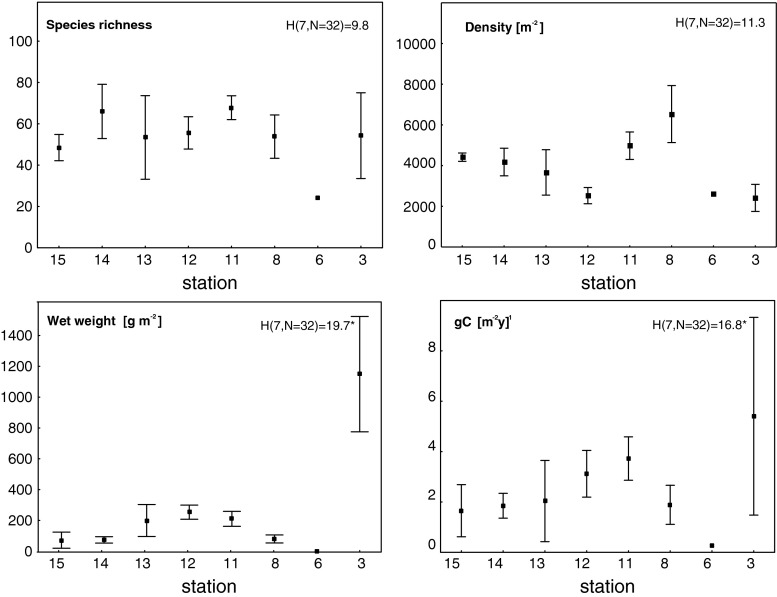
Mean infauna species richness, density [number of individuals per m^−2^], wet weight [g m^−2^], and secondary production [g C m^−2^year^−1^] for each station with 0.95 confidence intervals. Kruskal–Wallis results for differences between sampling sites are given with significant test results marked with *. Stations arranged from northwest (*left*) to southeast (*right*) on the *x*-axis

**Fig. 6:**
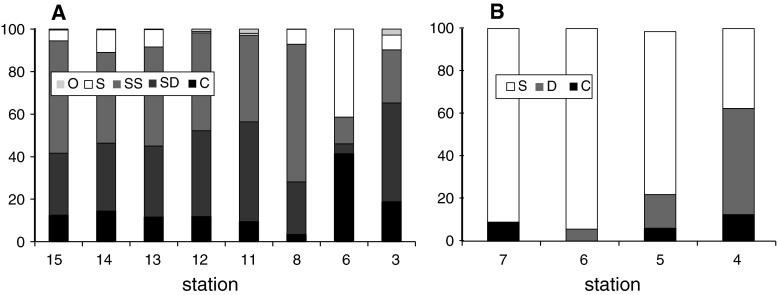
Percentage of different feeding guilds for *A* infauna and *B* epifauna. *O* omnivore, *S* suspension feeder, *SS* subsurface deposit feeder, *SD* surface deposit feeder, *D* deposit feeder, *C* carnivore. Station numbers are given under the horizontal axes

**Fig. 7:**
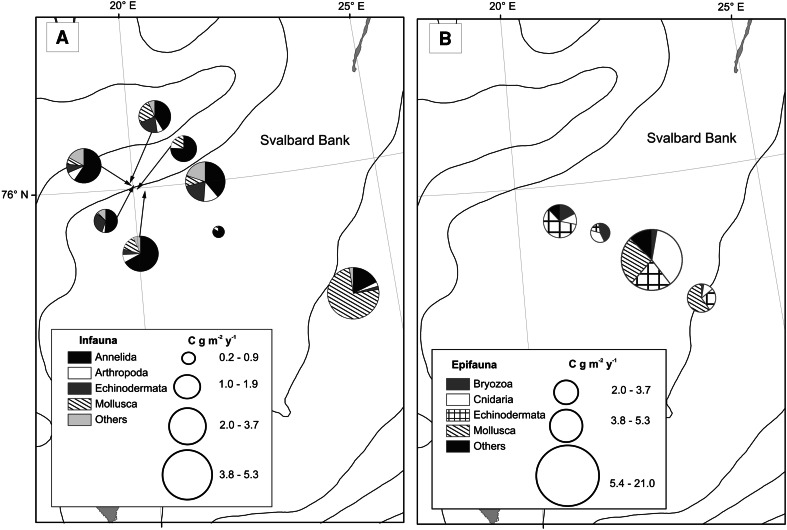
Mean production [g m^−2^ year^−1^] for A—infauna and B—epifauna

The top of the bank was characterized by shell debris with stones and gravel, and as the depth increased (90–150 m), sediments were mainly mud-mixed gravel and stones (Table 1). Some depth-related differences in species richness, density, and wet weight and carbon values were observed for epifauna (station 4—the deepest, and stations 6 and 7, the shallowest at the top of the bank), while for infauna the pattern was not so obvious (Figs. 3, 4, 5). Stations under 100 m had rich epifaunal communities, and these communities were themselves quite different across the bank (ANOSIM test: R: 0.36, *p* < 0.05; all pairwise comparisons significant except for pair stations: 6 and 7; Figs. 3, 4). Epifaunal species richness, as determined from the video, was low and varied across the bank (Fig. 4), and the most abundant taxa visible in the video at the top of the bank were suspension feeders, with more deposit feeders occurring at the 95-m station (st. 4) where muddier sediments were found (Fig. 6). Station 5 (65 m) had significantly greater density and species richness than stations 6 and 7 (Kruskal–Wallis test and post hoc comparison, *p* < 0.05; Fig. 4). Epifaunal biomass reached a maximum at the top of the bank at station 7 (43 m depth) with an average wet weight of 1,436 ± 5,277 g m^−2^ (Fig. 4). At 95 m on the eastern edge of the bank top, the echinoid *Strongylocentrotus droebachiensis* and bivalve *Chlamys islandica* dominated in abundance and biomass (75 and 98 % of the species present, respectively; Table 2). At the shallower station 5 (65 m), the hydrozoan, *Hydrallmania falcata* and *Sertularia mirabilis*, and the echinoid, *S. droebachiensis,* were most abundant (with *S. droebachiensis* and the sea star *Crossaster papposus* dominating in terms of biomass; Table 2). There was a dense community of the bryozoans, *Eucratea loricata* and *Alcyonidium gelatinosum,* the sea cucumber, *Cucumaria frondosa*, and the hydroid, *Sertularia mirabilis*, at the very top of the bank (~40 m), with *C. frondosa* reaching highest biomass (55–89 %; Table 2). Epifaunal productivity varied from a minimum of 0.4 and maximum of 33.4 g C m^−2^ year^−1^ across the bank top and was highest (average: 21.8 g C m^−2^ year^−1^) at station 5 (65 m), with mainly mollusks, cnidarians, and echinoids contributing >75 %. At station 7 (43 m), sea cucumbers (*C. frondosa*) were responsible for over half of the secondary production (9.4 of 15.8 g C m^−2^ year^−1^, Fig. 7).

**Table 2:** Dominant species for infauna (IN) and epifauna (EPI), and percent of total abundance and biomass for sampling stations on the Svalbard Bank

Station	Depth [m]	Dominant species	% of total abundance	Dominant species	% of total biomass
3 (IN)	137	*Macoma calcarea*	12.3	*Macoma calcarea*	51.8
		*Leitoscoloplos mammosus*	9.2	*Hiatella arctica*	13.3
		*Lumbrineris mixochaeta*	8.6	*Ciliatocardium cilatum*	9.1
		*Paraonella nordica*	5.2		
Total			2412.5		1160.1
4 (EPI)	95	*Strongylocentrotus droebachiensis*	50	*Strongylocentrotus droebachiensis*	64
		*Chlamys islandica*	25	*Chlamys islandica*	34
Total			24.6		217
5 (EPI)	65	*Hydrallmania falcata*	34.9	*Strongylocentrotus droebachiensis*	66
		*Sertularia mirabilis*	13.8	*Crossaster papposus*	11
		*Strongylocentrotus droebachiensis*	13.7		
Total			61.2		327.1
6 (IN)	40	*Mysella dawsoni*	45.4	*Bylgides elegans*	58.5
		Nemertea	19.2	*Euspira* sp.	8.9
		*Crenella decussata*	17.3	*Mysella dawsoni*	8.4
				*Glycera lapidum*	7.4
				*Crenella decussata*	6.6
				Nemertea	5.7
Total			2600		7.5
6 (EPI)	40	*Eucratea loricata*	44	*Cucumaria frondosa*	54.9
		*Sertularia mirabilis*	25.5	*Eucratea loricata*	39
		*Alcyonidium gelatinosum*	13.1		
Total			9.2		272.7
7 (EPI)	43	*Cucumaria frondosa*	34.6	*Cucumaria frondosa*	88.6
		*Alcyonidium gelatinosum*	23.1		
		*Eucratea loricata*	11.5		
Total			15.2		1552
8 (IN)	100	*Leitoscoloplos mammosus*	27.1	*Astarte montagui*	25.8
		*Maldane sarsi*	11.5	*Yoldia hyperborea*	13.6
		Euclymeninae	6	*Amphitrite cirrata*	13.3
				*Macoma calcarea*	9
Total			6532		79.7
11 (IN)	94	*Leitoscoloplos mammosus*	19.1	*Macoma calcarea*	33.2
		*Paraonella nordica*	8.4	*Strongylocentrotus droebachiensis*	21.3
		*Aphelochaeta* spp.	8.2	*Amphitrite cirrata*	12.5
		*Chaetozone* spp.	6.2	*Golfingia margaritacea*	5.9
		*Eteone* sp.	5.4		
Total			4976		206.5
12 (IN)	103	*Leitoscoloplos mammosus*	25.4	*Strongylocentrotus droebachiensis*	54
		*Paraonella nordica *	7	*Golfingia margaritacea*	15.6
		*Aphelochaeta* spp.	6.3	*Macoma calcarea*	7.2
Total			2524		274.95
13 (IN)	113	*Leitoscoloplos mammosus*	19.2	*Ciliatocardium cilatum*	51.9
		*Galathowenia oculata*	7.4	*Strongylocentrotus droebachiensis*	15.5
		*Anobothrus gracilis*	6	*Henricia* sp.	13.8
		*Lumbrineris mixochaeta*	6		
		*Paraonella nordica*	5.7		
Total			3666		208.13
14 (IN)	133	*Leitoscoloplos mammosus*	14	*Golfingia margaritacea*	26
		*Galathowenia oculata*	9.9	*Henricia* sp.	17.5
		*Lumbrineris mixochaeta*	8.6	*Ascidiacea*	9.7
		*Chaetozone* spp.	7.2		
Total			4176		81.92
15 (IN)	150	*Galathowenia oculata*	20.2	*Henricia* sp.	67.4
		*Maldane sarsi*	10.5	*Nepthys ciliata*	5.8
		*Leitoscoloplos mammosus*	10.2		
		*Lumbrineris mixochaeta*	9.9		
		*Chaetozone* spp.	8.8		
		*Chaetozone setosa* agg.	5.4		
Total			4415		76.98

Species that made more than 5 % of total abundance and biomass are listed for infauna and more than 10 % for epifauna. Total infaunal and epifaunal abundance [ind. m^−2^] and biomass [wet weight g m^−2^] are given for each station

Infauna dominated at bank slopes, in the deeper, muddier sites at depths greater than 100 m (Fig. 5), and significant differences in the community structure were found among different locations across the bank (ANOSIM test: R: 0.79, *p* < 0.05; all pairwise comparisons were significant except for station pairs: 13 and 14, and all pairs with station 6 and 15; Fig. 3). Biomass (wet weight) was the highest at the east slope (station 3, 1,160 ± 748 g wet weight m^−2^), below 260 g m^−2^ at most of the other stations, and below 10 g m^−2^ in the single grab at station 6 on top of the bank (Fig. 5). Species richness and density varied across the bank, but no significant differences were found among stations (Kruskal–Wallis test; *p* > 0.05); however, significantly higher biomass and production were found at the east slope of the bank (Kruskal–Wallis test; *p* < 0.05; significant differences (post hoc test) between station 3 and 8, and 3 and 14; Figs. 5, 7). Here mollusks occurred in high numbers, while the western slope was mainly occupied by polychaetes (Table 2; Fig. 7). Most of the infaunal species were surface deposit- and subsurface deposit-feeding polychaetes except from the top of the bank where carnivores and suspension feeders dominated (Fig. 6). Infaunal productivity varied 0.01 g C m^−2^ year^−1^ at the top of the bank and 1.1–3.7 g C m^−2^ year^−1^ on the sides and was highest at the eastern side with mollusks contributing 3.8 of total 4.3 g C m^−2^ year^−1^, Fig. 7).

## Discussion

### Faunal patterns

There was considerable variability in community structure and function across the bank that corresponded with differences in both the physical structure of the habitat and prevailing water masses. There were significant differences between epifaunal species richness, density, wet weight, and productivity across the stations as well as in infaunal biomass and productivity. In the Barents Sea, benthic faunal composition is highly dependent on the predominating water masses (Arctic or Atlantic), bottom substrate, and depth, generally as they impact carbon supply for food-limited communities (Carroll et al. 2008; Cochrane et al. 2009). Sediment type, adequate attachment sites, and bottom current flows are important factors for megafaunal distribution (Piepenburg et al. 1995) and also appear to be important in defining habitat structure over short distance and depth differences across the bank. Moreover, the physical dynamics can play an important role in community function with fauna shifting to suspension feeders in dynamic areas and deposit feeders in depositional areas (Feder et al. 2005, 2007).

The top of Svalbard Bank is characterized by very coarse substrate: mainly gravel, and mollusk and barnacle shells, while the areas below 90 m are covered with mud mixed with large stones and gravel. Strong residual currents over the central part of the bank (Kowalik and Proshutinsky 1995) continually resuspend recent, organic-rich sediments in these shallow areas. The coarse sediments at the top of the bank were largely dominated by suspension-feeding animals (cnidarians, bryozoans, and sea cucumbers) and surface predators (nemerteans, polynoid polychaetes). Since it is very difficult to sample infauna from the bank-top substratum, we have only a single grab from this area. This sample was full of shell debris, and although a full grab was taken and many small organisms (mainly suspension-feeding bivalve *Mysella dawsoni*) were found (2,600 ind. m^−2^), their contribution to the total community biomass was negligible (below 10 g m^−2^). Similar results were obtained from the few bank stations sampled by Cochrane et al. (2012), who found mainly sessile suspension feeders and motile carnivores at the depths of 60 m of the Svalbard Bank. The predominance of suspension feeders at the shallow part of the bank is consistent with strong, turbulent particle-rich currents, likely providing high food input to the seafloor and a high level of resuspension of organic material. The low representation of burrowing fauna on the shallow bank is consistent with the thin layer of surface sediment present (this study).

The percentage of surface and subsurface deposit feeders increased with depth and increasing proportion of mud in the sediment. The percentage of these feeding groups exceeded 60 % below 90 m for both epi- and infauna. On the eastern side (station 3: 115 m), burrowing polychaetes and bivalves, and carnivorous taxa such as polychaetes (*Pholoe* spp.*, Nothria conchylega*) and brittlestars (*Ophiura robusta*) were observed in this study and by Cochrane et al. (2012). The polychaetes *N. conchylega* and *Thelepus cincinnatus* were found in relatively high numbers on the banks of the Northeast Water Polynya in the Greenland Sea (Piepenburg et al. 1997), and *T. cincinnatus* has been noted as characteristic of undisturbed bank habitats (Hermsen et al. 2003). We did not find *T. cincinnatus* in our samples, but it was abundant in some of the same stations at the study of Idelson (1930).

The infaunal biomass ranged between 79.7 and 275.0 g ww m^−2^ (and 7.5 g ww m^−2^ at the top of the bank) which is similar to other Arctic localities (Table 3). However, infauna prevailed only at the sites where a significant percentage of mud was present, as also noted for the Chukchi Sea infauna (Feder et al. 1994, 2007), while epifauna dominated on the top of the bank. Epifaunal biomass ranged from 217 to 1,552 g ww m^−2^ which is higher than other Arctic locations (Table 3).

**Table 3:** Summary of epi- and infauna biomass (average g m^−2^ wet weight) for different locations in the Arctic

Area	Depth	Epifauna	Infauna	References
Barents Sea	199–503		10–152	Carroll et al. (2008)
North of Svalbard	132–510		43.2–253.4	Carroll and Ambrose (2012)
Chukchi Sea	<60	1.6–217.0	4.6–1420 (4000 in Barrow Canyon)	Grebmeier et al. (2006), Feder et al. (1991, 2007), Bluhm et al. (2009)
Laptev Sea	14–45	15.95		Piepenburg and Schmid (1997)
Northern Bering Sea			300–400	Grebmeier et al. (2006)
Beaufort Sea	32–90	0.27–81.22*		Renaud et al. (2007)
Beaufort Sea			200–680	Dunton et al. (2005)
Svalbard Bank	40–150	217–1552	79.7–275.0	This study

* Minimum biomass (only ophiuroids and sea urchins included)

Ophiuroids are among the most abundant megafauna on Arctic continental shelves (Piepenburg 2000). They occur in high densities on bank areas of the Barents Sea (Piepenburg and Schmid 1996a), as well as on other shallow shelf areas including Laptev, Beaufort and Chukchi Seas, and northeast Greenland (Piepenburg and Schmid 1996b, 1997; Piepenburg et al. 1997; Ambrose et al. 2001; Feder et al. 2005; Grebmeier et al. 2006; Renaud et al. 2007; Bluhm et al. 2009; Cochrane et al. 2012). Our data are consistent with these findings (ophiuroid densities about 150 ind. m^−2^ at the eastern slope, below 100 ind. m^−2^ on the west side). Densities of other echinoderms were found to be similar or more abundant in the more shallow areas, including sea cucumbers on the top of the bank and sea urchins on the eastern side. Ophiuroids are probably not able to resist strong currents in these areas, in contrast to the heavy sea cucumbers and sea urchins that were present (Jewett et al. 1999). The absence of ophiuroids in the shallowest waters of bank habitats was noted in the Laptev Sea (Piepenburg and Schmid 1997). *Ophiura sarsi* in the southeastern Chukchi Sea was also absent or uncommon in shallow water and abundant in deeper water (Feder et al. 2005).

Benthic faunal densities are likely related to large-scale water-column processes that determine food availability (Ambrose et al. 2001; Piepenburg 2000; Grebmeier et al. 2006), and benthic assemblages often reflect different hydrographic regimes and advective processes (Piepenburg et al. 1997; Feder et al. 2005, 2007; Carroll and Ambrose 2012). In the Barents Sea, sea ice, water masses, and primary production impact faunal distribution and density (Cochrane et al. 2009). We found significant differences in infaunal species composition, biomass, and productivity between eastern and western sides of the Svalbard Bank, which are likely due to different habitat properties including currents, sediment, and water-column properties. The station on the eastern side had the highest infaunal biomass (1,160 g m^−2^) in the sampled area with a relatively low productivity (4.37 g C m^−2^ year^−1^), mainly due to a high density of suspension-feeding mollusks, including *Hiatella arctica*, *Macoma calcarea,* and *Ennucula tenuis*. Studies of the Northeast Water Polynya in Greenland show that densities and composition of infauna and epifauna are largely influenced by dynamics of the overlying water column (Piepenburg et al. 1997). This demonstrates the importance of mesoscale pelagic processes in food providing and thus the importance of benthic pelagic coupling for benthic communities patterns. The circulation regime, sea-ice dynamics, and pelagic productivity can have a profound impact on benthic fauna. In our study, the eastern side of the Svalbard Bank is under the influence of the Polar Front with cold Arctic Water, while in the western part warm Atlantic waters prevail. The latter waters in this area are known to be two times more productive in phytoplankton (av. 90 g C m^−2^ year^−1^ in contrast to Arctic Waters <40 g C m^−2^ year^−1^) (Sakshaug and Slagstad 1992). The primary productivity of the water over the eastern side of Svalbard Bank contradicts our findings on benthic biomass. However, even if the eastern side does not have high water-column productivity like the western side, the rich fauna there may be supported by large amounts of particulate organic carbon (POC) brought in by the stronger currents to the abundant suspension feeders present. High benthic biomass may also suggest different sedimentation regimes and resuspension rates on the east and west side of the bank.

There was a distinct peak of epifaunal density at 60 m depth where a well-mixed relatively warm and low-saline water mass is present with currents that are not as strong as on the bank top. The highest biomass (station 7; 1,552 g m^−2^) was at the top of the bank, while productivity (avg. 21.8 g C m^−2^ year^−1^) was the highest at the depth of 65 m. The shallow water above the bank is well mixed due to winds and tides, providing considerable amounts of fresh organic matter and resuspended material for resident fauna (Fer and Drinkwater 2012). This food supply, however, is only available to suspension feeders able to withstand the high currents on the shallow bank top. This pattern is different from the one reported from the Beaufort Sea where epifaunal density and biomass were highest at the depth range of 60–90 m, with sharp declines in both shallower and deeper areas. The shallow waters in latter study were characterized by significant ice scour and high sediment discharge from the Mackenzie River (Renaud et al. 2007).

Shallow banks in the Barents Sea, even though recognized as important diversity and productivity “hot spots,” remain largely understudied in terms of species composition and function. Difficulties with quantitative sampling in areas where coarse substrate and high currents dominate most likely lead to this undersampling. Many Arctic shelves have complex bathymetries with shallow banks and deeper trough areas, including shallow platforms of the Chukchi and Greenland Seas, and very shallow (<20 m) banks in the Laptev Sea. Most of the shallow banks are characterized by rich and diverse epifaunal communities and high primary productivity compared to the deeper areas (Grebmeier et al. 2006; Piepenburg and Schmid 1996a, 1997; Piepenburg et al. 1997), and even though there are some exceptions (e.g., Beaufort Sea; Renaud et al. 2007), the fauna of the Svalbard Bank follows this pattern. In deep parts of the Barents Sea, benthic biomass can reach on average of about 100 g m^−2^ wet weight (Gulliksen et al. 2009), while on the bank it was on average about 600 g m^−2^ and maximum of over 3,500 g m^−2^ (this study, Idelson 1930). It confirms the considerable importance of this shallow bank in overall Barents Sea productivity.

### Temporal patterns: evidence for recovery of benthic communities?

Our results are consistent with the results obtained from similar station locations on the Svalbard Bank occupied approximately 85 years ago by Idelson (1930). Not only is the species composition described by Idelson (1930) similar to that found in our study, but also most of the species have similar densities and biomass. These results are surprising considering the heavy fishery activities (Gulliksen et al. 2009) and some significant climate and faunal shifts that have taken place since the 1920s (Blacker 1965; Drinkwater 2006).

*Chlamys islandica* was heavily fished in western Barents Sea from 1987 until 1992 when fisheries collapsed due to depletion of the scallop stock. Dredging and trawling activities can have different impacts depending on the habitat type, but can cause a significant decrease in biomass of epifaunal species such as sponges, hydroids, soft corals, bryozoans, and echinoderms dominant in our study (Kaiser et al. 2002; Lokkeborg 2005; Boulcott and Howell 2011). Since many of these taxa are characterized by long life spans (e.g., *C. islandica* can live up to 25–30 years (Gulliksen et al. 2009), sea urchins up to 42–75 years (Bluhm et al. 1998)), they are likely sensitive to anthropogenic and natural disturbance, and overfishing, especially since many need years to achieve a reproductive age (e.g., *C. islandica* at the age of 3.5 years).

Twenty years after cessation of heavy fishing on the Svalbard Bank, our results show that the benthic communities in the Svalbard Bank area are recovering from the damage they sustained. Collapsed stocks of scallops are apparently recovering, although the densities (2 ind. m^−2^) and biomass (12 g m^−2^) in our study are lower than in 1920s (average 10 ind. m^−2^, 467 g m^−2^; Idelson 1930). Since there are no records of sampling at these sites immediately following scallop harvesting, we cannot be certain that this transect was targeted by fishers or to what effect it experienced reductions in epifaunal densities. This area, however, is very accessible (no major obstructions such as coral reefs that trawlers would avoid) and had significant quantities of harvestable scallops, and trawlers are quite efficient at exploiting available resources. Thus, although our interpretation of system recovery is speculative, it is likely that 20 years may be a sufficient time for the shallow Svalbard Bank fauna to initiate recovery from trawling and dredging.

Studies on the recovery rate of benthic populations in other fished areas (e.g., Georges Bank) showed a steady and marked increase in the production of the benthic megafauna after 5 years since cessation of trawling and dredging activities by fishers (Hermsen et al. 2003). Deep sites, unlike the shallow areas, seemed to be greatly affected by fishing disturbance (Hermsen et al. 2003). Atlantic sea scallops (*Placopecten magellanicus*) and sea urchins dominated production at the recovering shallow sites, while deeper disturbed areas were characterized by the bivalves *Astarte* spp. and *P. magellanicus*. The polychaete *T. cincinnatus*, present on Svalbard Bank, made only a small contribution to the fauna in disturbed shallow sites on Georges Bank. On the contrary, it dominated in the deeper undisturbed areas. This species was present on Svalbard Bank in 1920s (Idelson 1930), but absent from the present study. This suspension-feeding soft-bodied tube-building polychaete was presumably unable to recover in contrast to bivalves which can survive in the wake of a scallop dredge or fish trawl (Hermsen et al. 2003). It is likely that shallow high-energy areas recover more quickly or that fauna living there is more resilient as a result of their lifestyle.

In the 1920–1930s, there was a dramatic warming of the northern Atlantic Ocean, with warmer than normal sea temperatures, that reduced sea ice conditions and increased input of Atlantic waters into the Barents Sea. This resulted in northward expansion of boreal species including benthic invertebrates and fish species, while Arctic species retreated northward (Blacker 1965; Drinkwater 2006). The Barents Sea appears to be exposed to cycles of “warm” years with large northward transports of heat and “cold” years (Loeng 1991), and there has been a warming trend over the past 10–15 years. Most of the species living on the Svalbard Bank are of boreal or of arcto-boreal origin, which means that they can adjust and even benefit from warmer conditions. Moreover, changes in fisheries pressures might be even more important for the biomass fluctuations than the temperature (Denisenko 2007). This may be the reason why, despite large climatic shifts over the last 85 years, little change was observed in the current study.

### Productivity and carbon cycling patterns

Shallow banks in the Barents Sea may play a disproportionally large role in this ecosystem since the primary production in these areas is about 2–3 times higher than in the adjacent, deeper waters. Unlike these deeper areas, Svalbard Bank is continuously exposed to strong tidal currents that provide nutrients for continuous primary production throughout the period of the year with available sunlight. Maximum secondary (epifaunal) production was above 21 g C m^−2^ year^−1^ on the bank top (present study), while at sites between 110 and 150 m, infaunal production only averaged around 2.5 g C m^−2^ year^−1^. Assuming a liberal 30 % ecological efficiency (Piepenburg and Schmid 1997), this suggests a total epifaunal carbon demand of between 6 and 70 g C m^−2^ year^−1^. Although bacterial carbon demand may be considerable on some Arctic shelves (e.g., Renaud et al. 2007) and has been implicated to be important on Svalbard Bank (Węsławski et al. 2012), experimental studies show most benthic carbon cycling on Arctic shelves is related to the macro/megafauna (Clough et al. 2005; Renaud et al. 2007).

This has important implications for spatial patterns of carbon consumption and distribution of organic matter available for export. The high bank-community consumption and carbon demand are still considerably lower than the annual primary production, suggesting the possibility for considerable export of carbon to the deeper areas of the Barents Sea, even if planktonic consumption is around 50 % (Wassmann et al. 2006a). Despite large variability in benthic biomass and diversity in the Barents Sea, the carbon burial flux is relatively constant (19 ± 5 mg C m^−2^ day^−1^) regardless of location or bloom stage (Reigstad et al. 2011). A new modeling study indicates that carbon deposition in shallow areas of Svalbard Bank is likely zero as any material reaching the sea floor is rapidly resuspended (Ellingsen et al. in prep). Advection of organic material from areas of high production has been shown to enhance distant benthic communities (Grebmeier et al. 2006) and fisheries dependent on benthos (De Leo et al. 2010). However, further work is required to balance the Svalbard Bank carbon budget, and ecological models are perhaps the best method for achieving this goal. Considering the commercial importance of benthic invertebrates and demersal fish fauna in the Barents Sea (e.g., Johannesen et al. 2012), carbon enhancement from highly productive banks can play an important role for local fisheries.
